# Anti-cholinesterase and Neuroprotective Activities of Sweet and Bitter Apricot Kernels (*Prunus armeniaca* L.) 

**DOI:** 10.22037/ijpr.2019.15514.13139

**Published:** 2020

**Authors:** Yasaman Vahedi-Mazdabadi, Elahe Karimpour-Razkenari, Tahmineh Akbarzadeh, Hania Lotfian, Mohammad Toushih, Neda Roshanravan, Mina Saeedi, Alireza Ostadrahimi

**Affiliations:** a *Nutrition Research Center, Tabriz University of Medical Sciences, Tabriz, Iran.*; b *Persian Medicine and Pharmacy Research Center, Tehran University of Medical Sciences, Tehran, Iran. *; c *Department of Medicinal Chemistry, Faculty of Pharmacy, Tehran University of Medical Sciences, Tehran, Iran. *; d *Electrophysiology Research Center, Neuroscience Institute, Tehran University of Medical Sciences, Tehran, Iran. *; e *Cardiovascular Research Center, Tabriz University of Medical Sciences, Tabriz, Iran.*; f *Medicinal Plants Research Center, Faculty of Pharmacy, Tehran University of Medical Sciences, Tehran, Iran.*

**Keywords:** Alzheimer’s disease, Amygdalin, Cholinesterase inhibitors, Neuroprotectivity, P. armeniaca

## Abstract

Apricot (*Prunus armeniaca* L.) is a fruit cultivated in various parts of the world. Both sweet and bitter kernels of apricot have been used for the treatment of different diseases such as loss of memory in Iranian traditional medicine (ITM). In the present study, the inhibitory activity of sweet and bitter extracts of apricot kernels towards cholinesterase (ChE) enzymes, both acetyl and butyrylcholinesterase was examined through Ellman’s method. In addition, neuroprotectivity of aqueous extracts and amygdalin were investigated against H_2_O_2_-induced cell death in PC12 neurons. Among them, the best acetylcholinesterase (AChE) inhibitory activity (IC_50_ = 134.93 ± 2.88 µg/mL) and neuroprotectivity (*P*-value < 0.0001) were obtained by the aqueous extract of bitter type. It was found that all extracts showed no butyrylcholinesterase (BChE) inhibitory activity.

## Introduction

Alzheimer’s disease (AD) is an age-related progressive neurodegenerative disorder characterized by the deterioration of memory leading to remarkable multilateral cognitive disorders with significant impact on the quality of life (1). It is also known as the most common cause of dementia in old age and according to statistics, the number of patients with dementia was 47 million in 2015 and it will reach 131.5 million by 2050 (2, 3). 

The reduction of the level of acetylcholine (ACh) in the cerebrospinal fluid, amyloid beta (βA) aggregation, irregular phosphorylation of tau proteins and subsequent formation of neurofibrils tangles in the hippocampal neurons, and oxidative stress have been involved in the pathogenesis of AD (4-7). 

Cholinesterase enzymes (ChEs) including acetylcholinesterase (AChE) and butyrylcholinesterase (BChE) belonging to the alpha/beta-hydrolase fold family have different tissue distribution (8). AChE is known to be found in neurons, whereas BChE mostly exists in plaques and tangles in glial cells (7). At present, the most promising therapeutic strategy is the inhibition of ChEs to prevent ACh neurotransmitter breakdown in neurons and synaptic clefts leading to the increased level of ACh in the basal forebrain (4). In addition, recent study reported by Mueller* et al.* revealed diminished mortality in patients with AD using AChE inhibitors (9). Furthermore, recent studies confirmed the efficacy of selective BChE inhibitors to promote the level of ACh and improve cognitive function (10). However, non-cholinergic role of ChEs by augmenting the formation of βA plaques has absorbed lots of attention (10-12). Hence, inhibition of ChEs not only improves the cholinergic function but can also be considered as an efficient strategy for preventing βA formation. Also, ChEs inhibitors have been recently considered for the cognitive improvements of dementia resulting from other neurological conditions such as Lewy body disease, Huntington’s disease, multiple sclerosis (MS), subcortical vascular dementia, and Parkinson’s disease (13, 14).

There is no definite treatment for AD and current four FDA-approved drugs including donepezil, rivastigmine, galantamine, memantine only ameliorate symptoms and the first three are AChE inhibitors (AChEIs). However, lack of significant efficacy and different side effects such as gastrointestinal disturbances, insomnia, fatigue or depression led to the conduction of a wide range of research to develop efficient anti-AD agents based on the multifactorial nature of disease (15). In this regard, various medicinal plants have been investigated for their effectiveness in treating AD and memory deficits. It is predicted that herbal agents regulate disease progression apart from symptomatic treatment (16-19). 

*Prunus armeniaca* L. known as apricot is a delicious stone fruit belonging to family Rosaceae, widely cultivated in the temperate regions such as Iran, Turkey, Pakistan, Iraq, Afghanistan, Syria, Russia, USA, Mediterranean countries, and central Asia. Among them, Iran and Turkey are the main producers in the world (20). In Iran, the fruit is grown throughout the country especially north-west where the climate is suitable for cultivating a highly desirable product. Apricot kernels contain various micronutrients and macronutrients such as fatty acids, sterol derivatives, polyphenols, carotenoids, volatile compounds, and cyanogenic glycosides (CNGs) (21). They are divided into two types of sweet and bitter kernels, depending on the amount of CNGs, mainly amygdalin (D-mandelonitrile-β-D-gentiobioside) which cause a bitter taste (22). They also have shown various pharmacological properties such as anticancer, antimicrobial, cardioprotective, hepatoprotective, antioxidant, anti-inflammatory, anti-amyloidogenic, neuroprotective, and neurotrophic activities (21, 23-25). Moreover, apricot kernels were recommended for the treatment of dementia and promotion of the memory in ITM (26).

In this study, according to our previous study on herbal anti-ChE agents and focusing on the efficient biological activity of apricot kernels which were supported by both classic medicine and ITM, anti-ChE and neuroprotective activities of different sweet and bitter apricot kernels’ extracts from north-west of Iran were investigated (27, 28). 

## Experimental

*Plant materials*

The fresh fruits (*Prunus armeniaca* L.) were collected from Ivand, a village in East Azerbaijan with coordinates (38°20′57″N, 46°07′01″E, at 1632.7m altitude above sea level) on 6 July 2016. They were fetched to the laboratory and the stones were removed. Then, they were identified by Prof. G.R. Amin and the voucher specimens PMP-750 and PMP-768 were deposited in the Herbarium of Faculty of Pharmacy, Tehran University of Medical Sciences, Tehran, Iran for sweet and bitter types, respectively. 

*Extraction*

The sweet and bitter apricot kernels were shade-dried and then milled using a mortar and pestle, given codes **S** and **B**, respectively. 

Petroleum ether extracts **SO** and **BO**: to extract fatty oils, 600 g of **S** and 130 g of **B** were separately macerated three times, each time with 3100 mL and 1100 mL petroleum ether, respectively for 72 h at room temperature. The solvent was concentrated under vacuum and the resulting oils (**SO** and **BO**) were stored at 20 °C. Also, the resulting residue in this step was dried at room temperature to give 342 g and 70 g solids **S1** and **B1**, respectively which were used for further extractions including hydroalcoholic, ethanolic, and aqueous extractions. 

Hydroalcoholic extracts **HS1** and **HB1**: 300 g of **S1** and 50 g of **B1** were separately extracted three times with methanol-water (70:30 (v/v) with total volume (3700 mL) and (970 mL) respectively, by maceration for 72 h at room temperature. The extracts were filtered and centrifuged at 4000 rpm for 6 min (Heraeus Megafuge 1.0, England), concentrated using a rotary evaporator under vacuum (Heidolph, Heizbad Hei-VAP, Germany) at 20 °C, and finally freeze-dried (LTE science LTD, England) at 60 °C/10 μmHg for 24 h to obtain desired extracts **HS1** and **HB1** in 16.30% and 22.74%, respectively which were stored at 20 °C.

Aqueous extracts **AS1** and **AB1**: 10 g of **S1** and 10 g of **B1** were separately boiled with 150 mL distilled water in a beaker for 10 minutes. Then, they were cooled and filtered. Each solid residue was re-extracted by 50 mL distilled water. They were filtered, centrifuged at 4000 rpm for 6 min, concentrated using a rotary evaporator under vacuum, freeze-dried, and stored at 20 °C to afford desired extracts of **AS1** and **AB1** in 27.5% and 24.2%, respectively.

Ethanolic extracts **ES1** and **EB1**: 10 g of **S1** and 10 g of **B1** were separately extracted three times with 245 mL ethanol (96%) by maceration for 72 h at room temperature. Next, the collected solvents were filtered, centrifuged at 4000 rpm for 6 minutes, concentrated using a rotary evaporator under vacuum, freeze-dried to yield 7.3% and 6.9% **ES1** and **EB1**, respectively which were stored at 20 °C. The same procedure was performed for **S** and **B** to afford ethanolic extracts of **ES** (13.2%) and **EB** (9.3%).

*In* *-* *vitro AChE and BChE inhibition assays*

Anti-AChE and anti-BChE activity of all extracts as well as amygdalin were investigated using modified Ellman’s method (29). 

Electric eel (Torpedo Californica) AChE (E.C. 3.1.1.7, type VI-S, lyophilized powder), BChE (E.C.3.1.1.8, from equine serum), acetylthiocholine iodide, butyrylthiocholine iodide, 5, 5′-dithiobis (2-nitrobenzoic acid) (DTNB), amygdalin and rivastigmine were purchased from Sigma (Steinheim, Germany).

10 mg of aqueous extracts of *P. armeniaca* was dissolved in 1 mL distilled water and the other extracts were dissolved in a mixture of 50% DMSO and 50% methanol to obtain dilution of stock 10 mg/mL and afterwards four different concentrations of each extract were examined to get enzyme inhibition for AChE/BChE. In the case of amygdalin, the stock solution was prepared by dissolving 1 mg of that compound in 1 mL distilled water. In a 96 well plate, 50 µL phosphate buffer (0.1 M, pH 8.0), 25 µL of the sample solution, DMSO/MeOH or distilled water and 25 µL of enzyme [0.22 U/mL of AChE or BChE] was added. The plate was pre-incubated for 15 min at room temperature. Then, 125 µL of DTNB (3 mM in buffer), as well as 25 µL of substrate (3 mM in water) (acetylthiocholine iodide or butyrylthiocholine iodide) was added to each well. Finally, the change of absorbance was recorded at 405 nm after 20 min using a microplate reader (BioTek ELx808, USA). 

Rivastigmine was applied as the positive control, while a mixture including buffer, DMSO, DTNB, and substrate was tested under the same conditions without enzyme as the negative control. 

*Neuroprotection study assays*

Rat pheochromocytoma PC12 cell line was purchased from the Pasteur Institute (Tehran, IRAN) and culture media and supplements were purchased from Gibco (Paisley, UK). The cells were cultivated in DMEM supplemented with 10% fetal calf serum plus antibiotics (100 units/mL penicillin, 100 μg/mL streptomycin). To induce neuronal differentiation, PC12 cells were re-suspended using trypsin/EDTA (0.25%) and seeded in 96 well culture plate (4000cells/well) and cultured for 1 week in differentiation medium (DMEM + 2% h serum + NGF (100 ng/mL) + penicillin and streptomycin). To evaluate the effect of **AS1** and **AB1** as well as amygdalin on the survival rate of neurons, the culture medium was changed to NGF free medium and different concentrations of the above-mentioned extracts and amygdalin (1, 10, 100 μg/mL) were applied on cells. Quercetin (3 μg/mL) was applied as a positive control. The extracts and amygdalin were diluted in DMEM and added to each well in the volume of 10 μL. Then, after 3 h, induction of ROS mediated apoptosis was initiated by adding the H_2_O_2_ (400 μM) to their medium. After 12 h, MTT assay was performed (30). MTT solution (5 mg/mL) was added to each well in a volume of 10 μL, and 3.5 h later, 100 μL of the solubilisation solution (10% SDS in 0.01 M HCl (w/v) was added into each well. The plates were allowed to stand overnight in the incubator in a humidified atmosphere. Absorbance was measured at 570 nm with a reference wavelength of 630 nm using a plate reading spectrophotometer (BioTek ELx808, USA). Each experiment was performed in three replicates.

*Determination of total phenols content*

The total phenolic content of the aqueous extracts possessing anti-ChE activity was assessed using Folin-Ciocalteu assay (31). Folin-Ciocalteu (Steinheim, Germany) and gallic acid (GAE) (Darmstadt, Germany) were purchased from Sigma. The total phenolic content was expressed in gallic acid equivalents as mg per g extract. The calibration equation was (y = 0.0068x-0.0221) (R² = 0.9911) where y is the absorbance at 765 nm using an UV-Vis Spectrophotometer (Mecasys 2120uv, Korea) and x is the concentration of gallic acid mg/g extract.

*Determination of total flavonoids content*

Total flavonoid content in the aqueous extracts was determined by aluminum chloride colorimetric assay (32). Aluminum chloride and chatechin (CE) were purchased from Merk (Darmstadt, Germany) and Sigma (Darmstadt, Germany), respectively. Total flavonoid content of aqueous extracts was expressed as mg chatechin equivalents per g extract. The calibration equation was (y = 0.002x + 0.0227) (R² = 0.9975) where y is the absorbance at 510 nm and x is the concentration of chatechin mg/g extract. 

*Statistical* *analysis*

All experiments were accomplished in triplicates. The IC_50_ values were estimated graphically from log inhibitor the extract concentration versus percent of inhibition by using Microsoft Excel 2010 program. One-way ANOVA was used to assess significant differences among the treatment groups followed by Tukey’s multiple comparisons test that was carried out to determine the level of significance by GraphPad Prism 6 software (San Diego, CA, USA). It means statistically significant when *P* value was less than 0.05.

## Results

*In-vitro anti-AChE and anti-BChE activity of different extracts of P. armeniaca*

Anti-ChE activity of different extracts was evaluated comparing with rivastigmine as the reference drug (Table 1). Also, ChEI activity of amygdalin as one of the main constituents was investigated to demonstrate whether it is responsible for the corresponding inhibitory activity (33, 34) (Table 1).

As shown in Table1, **AB1** illustrated the best anti-AChE activity with IC_50_ = 134.93 μg/mL whereas **HB1**, **BO**, **EB1** and **EB** counterparts depicted no AChEI activity. Our results revealed that **AS1** showed moderate inhibitory activity against AChE (IC_50_ = 356.75 μg/mL) and the corresponding **HS1**, **SO**, and **ES** extracts showed low activity with IC_50_s = 418.79, 481.42, and 445.65 μg/mL, respectively. It was found that **ES1** and amygdaline showed no inhibitory activity against AChE and probably is not responsible for anti-AChE activity of apricot kernels. 

It should be noted that all extracts as well as amygdalin showed no anti-BChE activity.

*Neuroprotectivity against H*_2_ *O*_2_ *-induced cell death in PC12 neurons*

Aqueous extracts of **AB1** and **AS1** were selected for the investigation of *in-vitro* neuroprotective studies caused by H_2_O_2_ in differentiated PC12 neuron cells. 

**Figure 1:**
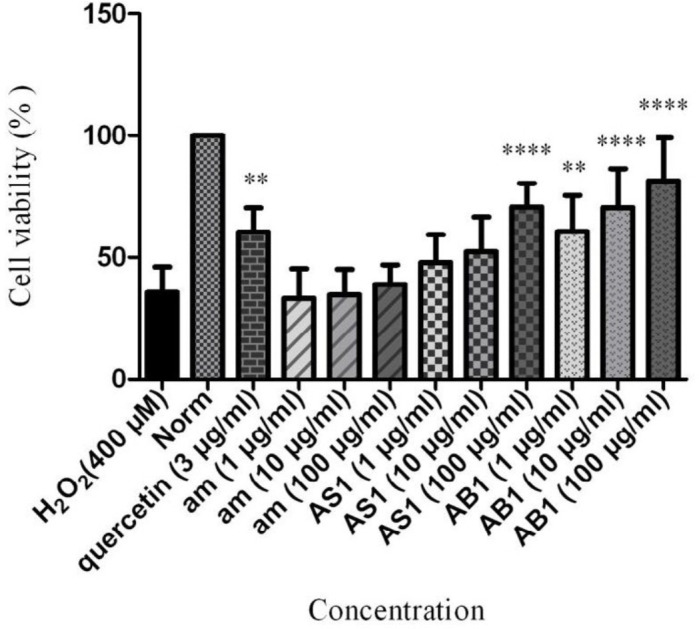
Neuroprotective effect of **AS1**, **AB1** and amygdalin on cell viability of PC12 cells in H_2_O_2_-induced damage. Data are expressed as mean SD and one-way analysis of variance (ANOVA) followed by Tukey’s multiple comparisons test was carried out to determine the level of significance. **P < 0.01, ****P < 0.0001 *vs*. control. am: amygdalin; **AS1**: aqueous extract of sweet apricot kernels; **AB1**: aqueous extract of bitter apricot kernels

Also, similar assay was conducted for amygdalin whether the neuroprotectivity was caused by amygdalin (Figure 1). As shown in Figure 1, the percentage of cell viabilities was measured at the concentrations of 1, 10, 100 µg/mL of those extracts in comparison to the H_2_O_2_-treated group as the control at the concentration of 3 µg/mL.

According to our results, PC12 cells pre-treated with **AB1** significantly protected neurons against H_2_O_2_ at 10 and 100 µg/mL (cell viability = 70.49 and 81.47% with *P*-value < 0.0001). However, it depicted moderate neuroprotectivity against cell death at 1 µg/mL (cell viability = 60.54% with *P*-value < 0.01). It was found that aqueous extract of **AS1** indicated remarkable neuroprotectivity at 100 µg/mL (cell viability = 70.71% with *P*-value < 0.0001). It exhibited no significant productivity at 1 and 10 µg/mL. Similar *in-vitro* evaluation of amygdalin at concentration of 1, 10, and 100 µg/mL showed no neuroprotectivity.

*Determination of total phenolic and flavonoid content*

Total phenolic and flavonoid content of aqueous extracts was reported in Table 2. As can be seen in Table 2, the extract of **AS1** contained more total phenolic and flavonoids content than **AB1** suggesting that AChEI activity is independent of phenolic and flavonoid compounds.

**Table 1:** The IC_50_ values of different extracts of *P. armeniaca* toward AChE and BChE

**Samples**	**AChEI [IC**_50 _ **(μg)/mL]**	**BChEI [IC**_50_ ** (μg)/mL]**
**AS1**	356.75 ± 0.171	> 500
**HS1**	418.79 ± 0.365	> 500
**SO**	481.42 ± 0.447	> 500
**ES**	445.65 ± 8.42	> 500
**ES1**	> 500	> 500
**AB1**	134.93± 2.88	> 500
**HB1**	> 500	> 500
**BO**	> 500	> 500
**EB**	> 500	> 500
**EB1**	> 500	> 500
Amygdalin	> 500	> 500
Rivastigmine	2.77 ± 0.09	1.93 ± 0.03

aData are expressed as Mean ± SE (three independent experiments).

**Table 2:** Total phenols and flavonoids content from aqueous extracts of *P. armeniaca*

**Sample**	**Total phenols** **(µg GAE/ mL)**	**Flavonoids** **(µg CE /mL)**	**Flavonoids/** **Phenolics**
**AS1**	21.33±0.06	16.07 ± 0.03	0.75
**AB1**	18.61±0.51	7.32 ± 0.17	0.39

## Discussion

AD is one of the most common types of dementia. In this respect, various synthetic and plant-derived ChEIs are usually used for the management and amelioration of the disease (15). In this study, *P. armeniaca* kernels were candidate to be examined for their anti-AD activities via ChEI and neuroprotective properties. The selection of kernels comes back to the efficacy of apricot kernels recommended for the prevention and treatment of memory loss in Iranian traditional medicine (ITM). For this purpose, a wide range of plants have been introduced in ITM which have been thoroughly investigated in the recent studies (27). Also, the presence of glycosides in the kernels may found to be suitable as they are one of the major classes of those compounds documented to possess ChEI activity (35, 36). 

It seems that the plants belonging to Rosaceae family containing CNGs have depicted anti-ChE activity (37, 38). According to the literature available, the concentration of constituents and biological properties of apricot kernels not only are affected by the kinds of apricot kernels (sweet or bitter) and solvent extraction, but also are affected by the variety, geographic region, and collection time (34, 39, 40). Hence, anti-ChE and neuroprotective activities of aqueous, hydroalcoholic, petroleum ether, and ethanolic extracts of both sweet and bitter kernels of apricot cultivated in north-western Iran were investigated. In addition, similar studies were conducted for amygdalin as the main constituent of kernels whether it is responsible for the desired activities. 

Our results revealed that aqueous extract of bitter apricot kernels showed potent *in-vitro* acetylcholinesterase inhibitory and neuroprotective effects. However, regarding to results reported in Table 1, amygdalin demonstrated no biological effects. Therefore, the observed effects probably are associated to the synergistic effects of several compounds. It is worth mentioning that AChEI activity of some plants from family Rosaceae including *Agrimonia eupatoria*, *Amygdalus scoparia*, *Coussapoa microcarpa*, *Cotoneaster nummularia*, *Rosa canina*, and *Rosa damascene *depicted low inhibitory activity and *P. armeniaca* was found much more potent than those reported in the literature (41). 

Another instructive point relates to the study reported by Cheng* et al.*, which revealed that amygdalin has improved nerve growth factor (NGF)-induced neuritogenesis and reduced 6-hydroxydopamine (6-OHDA)-induced neurotoxicity in rat dopaminergic PC12 cells (24). On the other side, prunasin known as CNGs which is ubiquitous in the apricot kernels has shown neuroprotective activity in NG108-15 hybridoma cells (42). The neuroprotectivity of bitter kernels may be related to the presence of flavonoids since these compounds have been found as effective neuroprotective agents (43, 44). For example, it has been revealed that the consumption of green tea led to the reduction of risk of Parkinson’s disease (43, 45). 

In a study reported by Tohda *et al.*, a kampo formula composed of *P. armeniaca* L., which is used for the improvement of learning impairment and synaptic loss in the patients with AD, was studied for its neuroprotective abilities in mice (46). It was found that synergistic effects played an important role in directly activating neurons.

There are various reports confirming that cyclooxygenase2 (COX-2) overexpression causes alteration of neuronal cell cycle in a murine model of AD and COX-2 inhibitors inhibited the cell-cycle in the early phase of βAP toxicity (47). Also, in a study reported by Yang* et al.*, amygdalin which was extracted from the seeds of *Prunus armeniaca* L. suppressed COX-2 and inducible nitric oxide synthase (iNOS) expressions in mouse BV2 microglial cells. It can be considered as an auxiliary role for the cognitive enhancement (48). However, it seems that further investigations are required to determine the exact mechanism of action of apricot kernels as an anti-AD agent. 

## Conclusion

In conclusion, *in-vitro* ChE inhibitory and neuroprotective activities of various extractions of bitter and sweet apricot kernels (*P. armeniaca* L.) were investigated. Our results demonstrated that the best anti-AChE activity and neuroprotectivity against H_2_O_2_-induced cell death in PC12 neurons were obtained by the aqueous extract of bitter type. To gain insight into more effective constituent responsible for the desired biological activities, amygdalin as the most abundant compound in the apricot kernels was similarly evaluated. As amygdalin showed neither ChE inhibitory nor neuroprotective activities the synergistic effects of multiple compounds are probably involved in inducing desired biological activities. Also, it is suggested that inhibitory activity of kernels extracts should be evaluated against different factors involved in the creation of AD such as βA aggregation, phosphorylation of tau proteins, and beta secretase (BACE1) to find the possible mechanism of action of apricot kernels for the treatment of dementia and improvement of memory to develop plant-mediated neurodegenerative drug discovery. 
